# Wound Healing Response After Bleb-Forming Glaucoma Surgery With a SIBS Microshunt in Rabbits

**DOI:** 10.1167/tvst.11.8.29

**Published:** 2022-08-26

**Authors:** Ralph J. S. van Mechelen, Jarno E. J. Wolters, Marjolein Herfs, Christian J. F. Bertens, Marion Gijbels, Leonard Pinchuk, Theo G. M. F. Gorgels, Henny J. M. Beckers

**Affiliations:** 1 University Eye Clinic Maastricht, Maastricht University Medical Center+, Maastricht, the Netherlands; 2 Department of Ophthalmology, School for Mental Health and Neuroscience, Maastricht University, Maastricht, the Netherlands; 3 Chemelot Institute for Science and Technology, Maastricht, the Netherlands; 4 Department of Pathology, Cardiovascular Research Institute Maastricht, School for Oncology and Developmental Biology, Maastricht University, Maastricht, the Netherlands; 5 Department of Medical Biochemistry, Experimental Vascular Biology, Amsterdam UMC, University of Amsterdam, Amsterdam, the Netherlands; 6 InnFocus, Inc., Miami, FL, USA

**Keywords:** glaucoma surgery, wound-healing response, animal model, microshunt, fibrosis

## Abstract

**Purpose:**

The PreserFlo MicroShunt is an innovative implant for the surgical treatment of glaucoma. Although usually effective, surgeries can still fail due to fibrosis. This study was conducted to gain insight into the histological aspects of the fibrotic response and find potential targets to reduce postoperative fibrosis.

**Methods:**

Fifteen New Zealand White rabbits were implanted with a microshunt and followed up for 40 days. Animals were euthanized at postoperative days (PODs) 1, 5, and 40 to collect eyes for histological evaluation. Bleb formation and ocular health were assessed by slit-lamp (SL) biomicroscopy and optical coherence tomography (OCT). Intraocular pressure (IOP) was measured using rebound tonometry.

**Results:**

Blebs failed after approximately 2 weeks based on bleb survival and IOP measurements. No severe complications were observed with OCT and SL. Histology revealed a wide variety of cells, in the bleb and around the microshunt, including polymorphonuclear leucocytes (PMNs), myofibroblasts, and foreign body giant cells, at different PODs.

**Conclusions:**

Implantation of a poly(styrene-*b*-isobutylene-*b*-styrene) microshunt in rabbits resulted in the occurrence of a wide variety of cells during the wound-healing response. Future research should further elucidate the potential of these (earlier often overlooked) cells to target the fibrotic response in vivo—for example, by developing novel antifibrotic drugs, methods for sustained delivery of medications, or augmenting material properties.

**Translational Relevance:**

Current antifibrotic therapies aim to inhibit myofibroblasts; however, a wide variety of cells are involved in the fibrotic response. Future research focusing on these cells could offer novel methods for reducing the fibrotic response after glaucoma surgery.

## Introduction

Glaucoma is classified as a degenerative optic neuropathy in which retinal ganglion cell loss and subsequent loss of optic nerve fibers lead to irreversible and progressive visual field loss.^1^^,^^2^ Untreated, the disease may lead to severe visual impairment and blindness. Currently, glaucoma is the leading cause of irreversible blindness worldwide. It is estimated that approximately 112 million patients will suffer from glaucoma in 2040.^3^ The primary risk factor is increased intraocular pressure (IOP). Currently, no causative treatment exists for glaucoma. The only proven treatment is lowering IOP to a target level that is sufficiently low to halt progression of the disease. Patients are primarily treated with medications (usually eye drops) and/or laser surgery to reduce IOP to “normal” limits (10–21 mmHg).^4^^–^^6^ Although highly effective, bleb-forming glaucoma surgery is often postponed as a last resort treatment due to vision-threatening complications.^1^ During glaucoma filtration surgery, IOP is lowered by creating a transscleral outflow channel through which aqueous humor (AqH) flows out of the eye into the subconjunctival/sub-Tenon's space. With this procedure, a small fluid-filled blister is formed, which is referred to as a filtering bleb.^1^^,^^7^ However, approximately 10% of all bleb-based surgeries fail each year due to excessive wound healing and the formation of fibrosis.^8^ Fibrosis limits the outflow of AqH by scarring of the bleb, leading to inadequate IOP reduction.^7^ Overall, fibrosis is considered as an overreaching wound-healing response, with myofibroblasts persistently present in the wound.^7^ To reduce the fibrotic response, the antimetabolites mitomycin-C (MMC) or 5-fluoracil are often used, either as a one-time application during surgery or as one or several injections into the bleb after surgery.^9^ Unfortunately, use of antimetabolites may be accompanied by complications such as endophthalmitis, corneal endothelial cell loss, avascular filtering blebs, hypotony, thinning of the conjunctiva, and bleb leaks.^10^ To improve the safety of glaucoma surgery, new and less invasive bleb-forming glaucoma surgical techniques have been introduced and commercialized over the last decade. One of these is the PreserFlo MicroShunt (InnFocus, Miami, FL), which is made from an innovative material, poly(styrene-*b*-isobutylene-*b*-styrene), or SIBS. In vivo research has previously shown that SIBS induces less encapsulation and activation of myofibroblasts when compared to silicone. Additionally, flow was still visible after 1 year of follow up.^11^ Clinical studies have shown that the overall success rate of the PreserFlo MicroShunt is 70% after approximately 1 year.^12^^,^^13^ Although effective in reducing IOP, surgeries may still fail due to the formation of fibrosis, even with the additional usage of MMC.^14^^,^^15^ Patients will often require additional IOP-lowering medications or surgeries to reach the desired low IOP level.

To find potential targets to further improve surgical outcomes, we wanted to investigate the wound-healing response after placement of a SIBS microshunt, on a histological level. For this purpose, we implanted rabbits with a SIBS microshunt and followed the histological response for up to 40 days. The SIBS microshunts that were used in our study are closely similar to the commercially available PreserFlo MicroShunt.

## Materials and Methods

### Animals

All animal procedures were approved by the Central Authority for Scientific Procedures on Animals, were approved by the local ethical committee, and were in accordance with the European Directive for animal experiments (2010/63/EU; approved Dutch license no. AVD1070020197464). They also complied with the ARVO Statement for the Use of Animals in Ophthalmic and Vision Research and the Animal Research: Reporting of In Vivo Experiments (ARRIVE) 2.0 guidelines. Fifteen normotensive New Zealand White rabbits (Charles River Laboratories, Evreux, France) were used, with an approximate age of 12 weeks and weighing 2.5 to 3 kg. Both male and female rabbits were used. The rabbits were maintained under controlled conditions of temperature and humidity on a 12-hour/12-hour light/dark cycle. The rabbits had ad libitum access to water and food (100 g of dried chow per rabbit per day and unlimited access to hay). Before the start of the experiment, animals had a 2-week acclimatization period. A randomized complete block design was used to allocate animals to groups, and animals were euthanized at postoperative days (PODs) 1, 5, and 40 (*n* = 5 per group) with an overdose of pentobarbital sodium, 200 mg/kg (Euthasol 20%; Produlab Pharma BV, Raamsdonksveer, Netherlands), intravenously injected.

### Surgical Procedure

**Figure 1.**
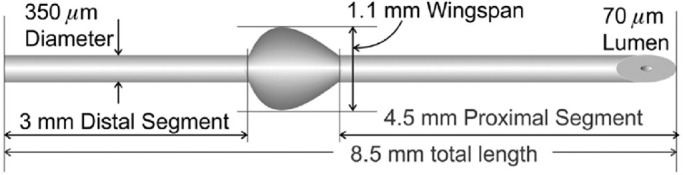
Schematic representation of the PreserFlo MicroShunt. The image is used with permission of InnFocus, Inc.

In total, 15 animals were randomly assigned to be implanted with a SIBS MicroShunt (Fig. 1). Animals were surgically anesthetized by injecting 30 mg/kg ketamine and 0.25 mg/kg medetomidine (Sedator; AST Farma BV, Oudewater, Netherlands) intramuscularly (IM). Additionally, inhalation anesthetics were provided if needed (0.5%–1% isoflurane). In all rabbits, the right eye was used for implantation. All surgeries were performed by a single surgeon. Local analgesia was provided in the form of 0.4% oxybuprocaine hydrochloride drops (Minims; Bausch & Lomb Pharmaceuticals, Brussels, Belgium). The surgical area was exposed with a speculum. A fornix-based conjunctival flap was created with Westcott tenotomy scissors, after which a 1-mm wide and 1-mm deep scleral pocket was formed, 3 mm posterior to the limbus. Through the scleral pocket, a needle tract was made with a 25-gauge needle into the anterior chamber. The microshunt was then implanted through the needle tract. The fins were wedged firmly into the scleral pocket. Afterward, flow was checked by massaging the eye until a droplet of aqueous formed at the distal end of the microshunt. Finally, the conjunctiva and Tenon's capsule were sutured with a Vicryl 9-0 suture (Ethicon, Inc., San Lorenzo, Puerto Rico). After completion of the surgery, the rabbit received an IM injection of 0.5 mg/kg atipamezole (Antisedan; Orion Pharma Animal Health, Espoo, Finland) to accelerate recovery. Animals were treated with 1% chloramphenicol ointment twice a day for 5 days and received a subcutaneous injection of buprenorphine (0.05 mg/kg Bupaq Multidose; Richter Pharma AG, Wels, Austria) up until POD 1 twice a day; if needed, analgesia was extended.

### In Vivo Measurements

Rabbits were sedated using 0.40 mg/kg medetomidine IM. Afterwards, rabbits were examined using IOP measurements with the iCare TONOVET (iCare Finland Oy, Vantaa, Finland). followed by slit-lamp (SL) biomicroscopy examinations (BI 900; Haag-Streit, Köniz, Switzerland); slit-lamp optical coherence tomography (SL-OCT; Heidelberg Engineering, Heidelberg, Germany), with the unit mounted on a Haag-Streit BD-900 SL; and bleb images taken with an EOS 4000D (Canon, Inc., Tokyo, Japan). One week before surgery, baseline recordings were taken for each rabbit. The follow-up examinations were performed on PODs 1, 5, 7, 11, 15, 25, and 40. When a bleb appeared to be flat, the eye was gently massaged; if the bleb remained flat after massage, the bleb was categorized as failed.

### Light Microscopy and Staining

**Figure 2.**
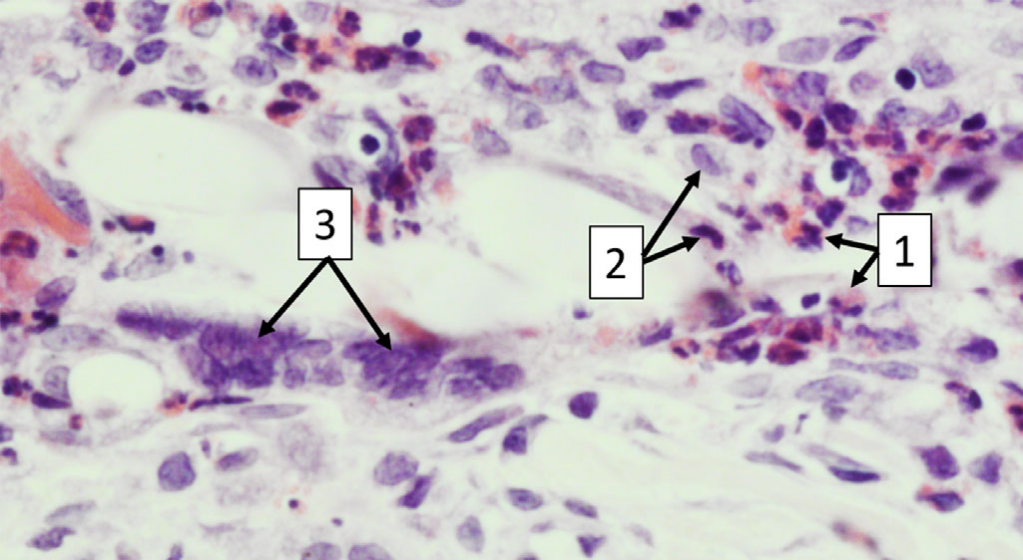
Examples of PMNs, macrophages, and foreign body giant cells (FBGCs). (1) PMNs are distinguishable due to their multilobed nucleus and eosinophilic cytoplasm. (2) Macrophages have a basophilic cytoplasm and a bean-shaped or ovoid nucleus. (3) FBGCs are large clusters of fused macrophages. The image was taken at 400× magnification.

Bromodeoxyuridine (BrdU, 30 mg/kg; Abcam, Cambridge, UK) was injected intravenously twice, one day before sacrifice. Animals were euthanized using 200 mg/kg pentobarbital sodium (Euthasol 20%). After euthanasia, both eyes were dissected and fixed in 4% paraformaldehyde (PFA) for 2 days (PFA was refreshed once a day); after fixation, eyes were dehydrated and embedded in paraffin blocks. Sections were cut with a thickness of 4 µm and were stained with hematoxylin and eosin (H&E), mouse anti-alpha-smooth muscle actin (α-SMA; MA5-11547, 1:500; Thermo Fisher Scientific, Waltham, MA) and sheep anti-BrdU (NB500-235, 1:2500; Novus Biologicals, Littleton, CO). In the case of anti-α-SMA and anti-BrdU, slides were blocked using 3% hydrogen peroxidase and 10% donkey serum (ab7475; Abcam). A secondary antibody, Donkey Anti-Mouse (715-065-151, 1:2000; Jackson ImmunoResearch, West Grove, PA), and Donkey Anti-Sheep (LS-C61150, 1:3000; LifeSpan Biosciences, Seattle, WA) with a biotin label was used to detect the primary antibody. Finally, a VECTASTAIN ABC-HRP kit (PK-6000; Vector Laboratories, Burlingame, CA) was used to enhance the biotin label for staining, and NovaRED (SK-4800; Vector Laboratories) was used to stain the slides. Histological slides were assessed and semiqualitatively scored by two blinded observers; as a control, the contralateral eyes were used (from the same time point as the experimental eye). Percentages of cells were graded by looking at high-magnification fields and estimating the number of cells present per field. Multilobed polymorphonuclear leucocytes (PMNs), which, in rabbits, have heterophilic cytoplasmic granules because they are heterophils,^16^ and macrophages were identified based on nuclear morphology, cytoplasm color, and nuclear cytoplasmic ratio (Fig. 2). Photographs were taken using a confocal scanning microscope (BX51; Olympus Corporation, Tokyo, Japan).

### Statistical Analysis

A two-way analysis of variance (ANOVA) with Sidak post hoc comparisons was used to compare the IOP between groups. A Kaplan–Meier survival curve was created to plot the bleb survival. Prism 9.3.1 (GraphPad Software, San Diego, CA) was used for the analyses.

## Results

### Bleb Morphology

**Figure 3.**
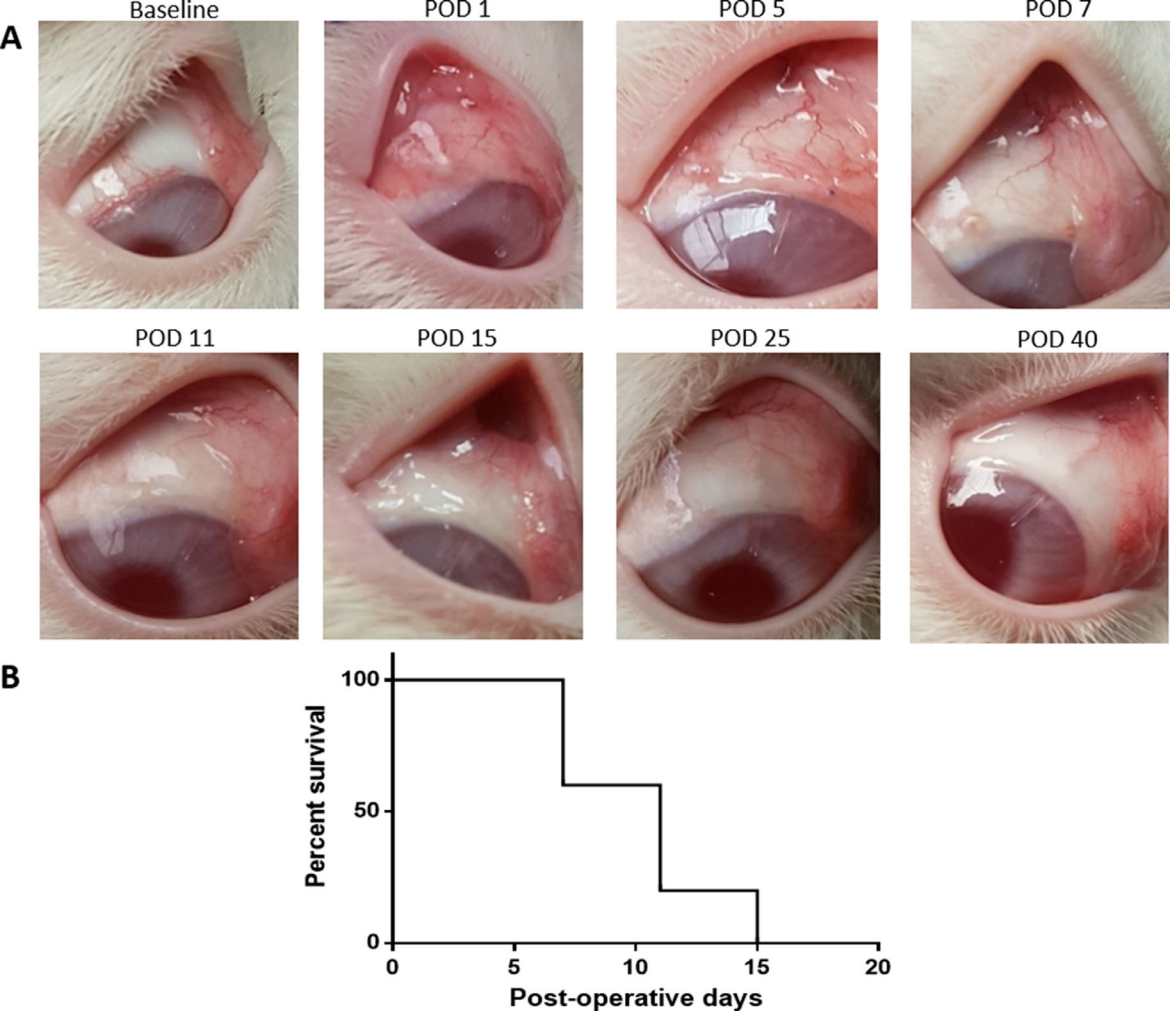
Overview of bleb morphology after microshunt implantation. (A) Baseline images showed a healthy eye without any signs of inflammation. After implantation, vascularization increased and a filtering bleb was visible. At POD 7, the bleb began to decline in height, becoming non-distinguishable at POD 15. At POD 25, the microshunt became visible through the conjunctiva, indicating that the tissue was being pulled back toward the sclera. At POD 40, signs of inflammation were no longer visible. (B) Bleb survival (*n* = 5) within 15 days postoperative, when all blebs appeared flat.

A detailed overview of the bleb morphology is provided in Figure 3. After surgery, blebs were well visible with increased signs of vascularization and redness that subsided around POD 7. All blebs appeared flat at POD 15. In some animals, at POD 40, the curvature of the microshunt could be seen through the conjunctiva due to contraction of the tissue (Fig. 3A). Bleb analysis (Fig. 3B) showed that all blebs failed within approximately 2 weeks postoperative; on average, blebs failed within 10.2 ± 2.99 days. No severe adverse events were noted. In several cases, corneal edema was visible with the OCT after surgery, which reduced to normal within 2 to 3 days (data not shown).

### Intraocular Pressure

**Figure 4.**
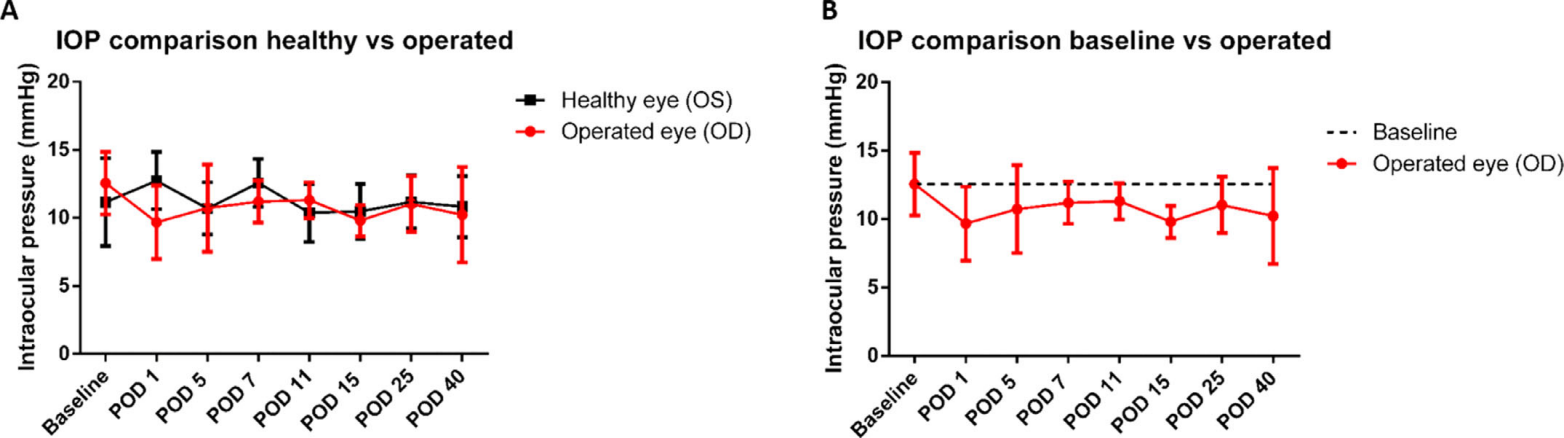
IOP measurements (*n* = 5). IOP measurements were performed at baseline and at PODs 1, 5, 7, 11, 15, 25, and 40. (A) Compared to the healthy eye, the IOP was reduced after surgery. A lower IOP was observed until POD 11. Afterward, no large differences between the healthy eye and operated eye were found. (B) The IOP remained below baseline (*dotted line*); no significant differences were found.

After surgery the IOP decreased, indicating a successful implantation and working filtering bleb. Although no significant differences were found between groups, a trend was observed showing that the IOP initially decreased at POD 1 and gradually increased again until POD 11 (Fig. 4A). At POD 11, the IOPs of both the operated and healthy control eyes were largely similar. Interestingly, IOP remained below baseline throughout the entire experiment (Fig. 4B).

### Histology

**Figure 5.**
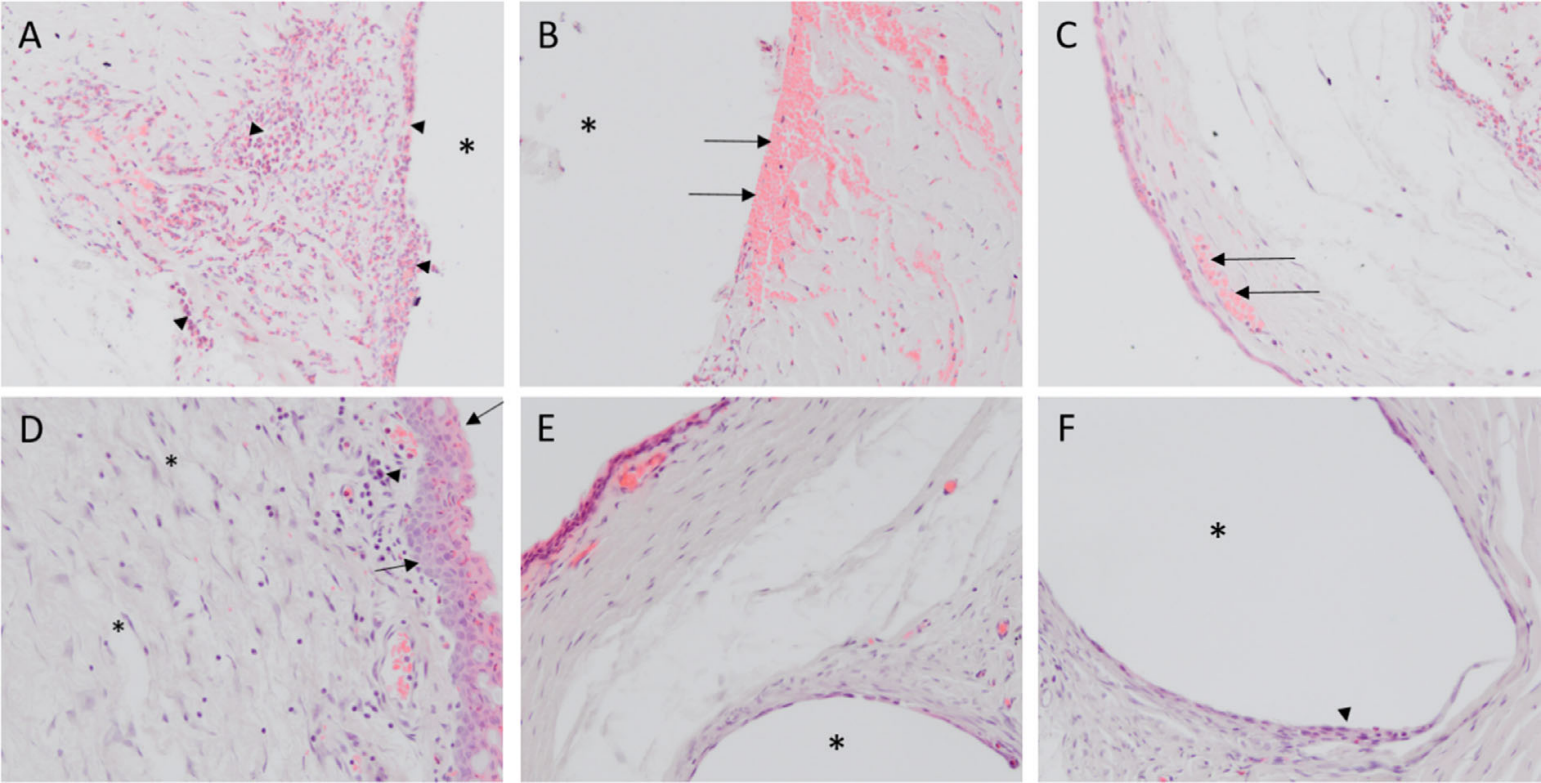
H&E staining at PODs 1, 5, and 40. (A) At POD 1, a high number of PMNs (*arrowhead*) had infiltrated the bleb area. (B) Red blood cells around the implantation site were clearly visible (*arrows*). (C) Some vessel swelling was visible around the conjunctiva (*arrows*). (D) POD 5 revealed significant granulation tissue in the bleb with many fibroblasts (e.g., asterisks) and macrophages (e.g., *arrowhead*) present. Additionally, an increased thickness of the conjunctiva was noted at this time point (*arrows*). (E) At POD 40, fewer inflammatory cells resided in the bleb; however, a low number of macrophages and PMNs were sporadically observed. The bleb wall contracted, and granulation tissue was replaced. (F) Although minimal, some foreign body giant cells are visible around the SIBS microshunt (*arrowheads*). *Position of SIBS microshunt. All images were taken at 200× magnification.

**Figure 6.**
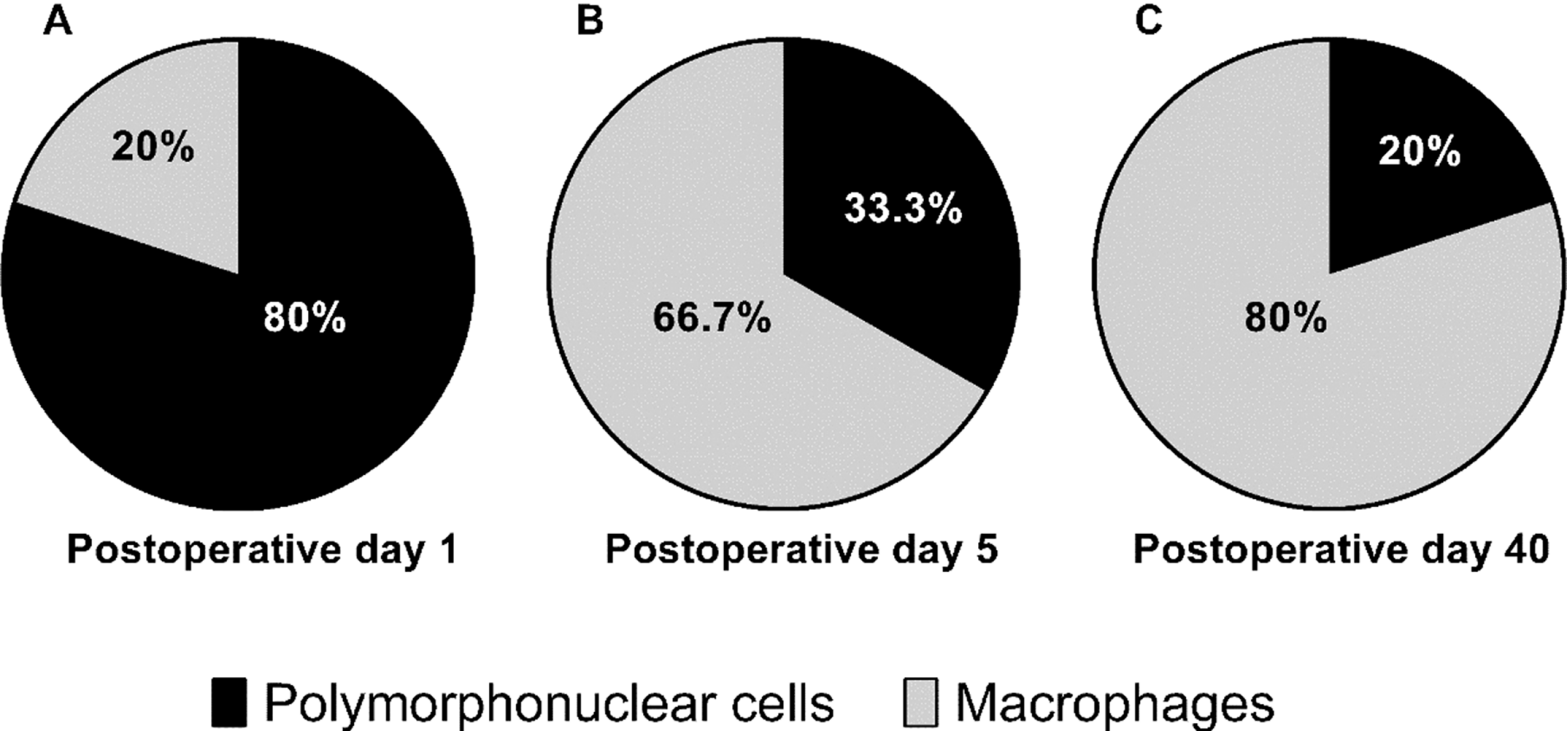
Grading of leukocytes present per POD in percentages. (A) On POD 1, an increased amount of PMNs was present (80%), indicating acute inflammation. (B) A shift in PMNs (33.3%) and macrophages (66.7%) was observed at POD 5. (C) At POD 40, a relatively high number of macrophages were still present in the bleb (80%), indicating a chronic inflammatory state.

**Figure 7.**
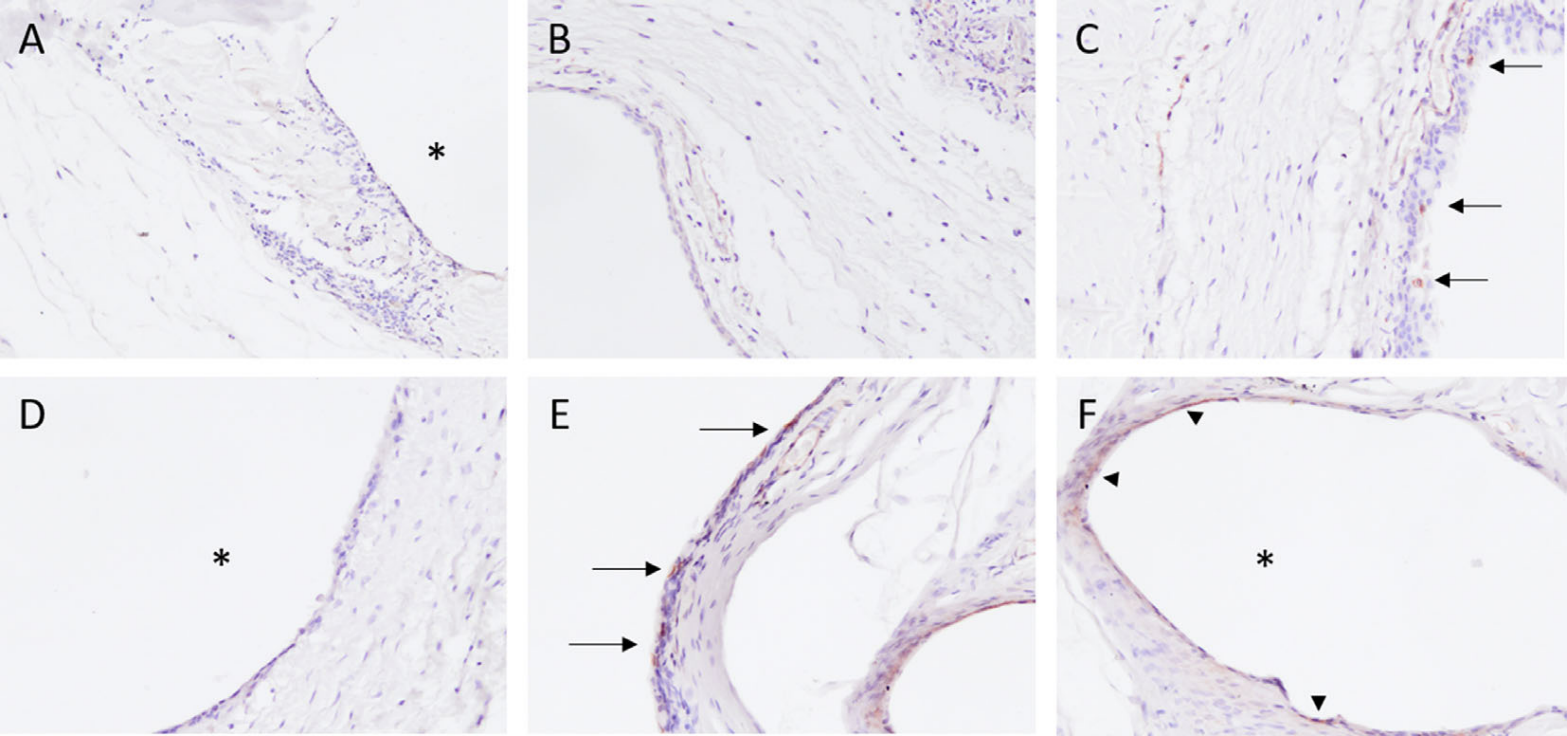
Representative images of the α-SMA. (A, B) No α-SMA was present at POD 1. (C) A limited amount of staining was visible at POD 5 in the conjunctiva (see *arrows*). (D) No α-SMA was present around the SIBS microshunt. (E) The α-SMA was mainly stained in the conjunctival wall at POD 40, indicating a strong presence of activated myofibroblasts at this time point (*arrows*). (F) Some staining was evident around the SIBS microshunt at POD 40 (*red arrowheads*). *Position of SIBS microshunt. All images were taken at 200× magnification.

**Figure 8.**
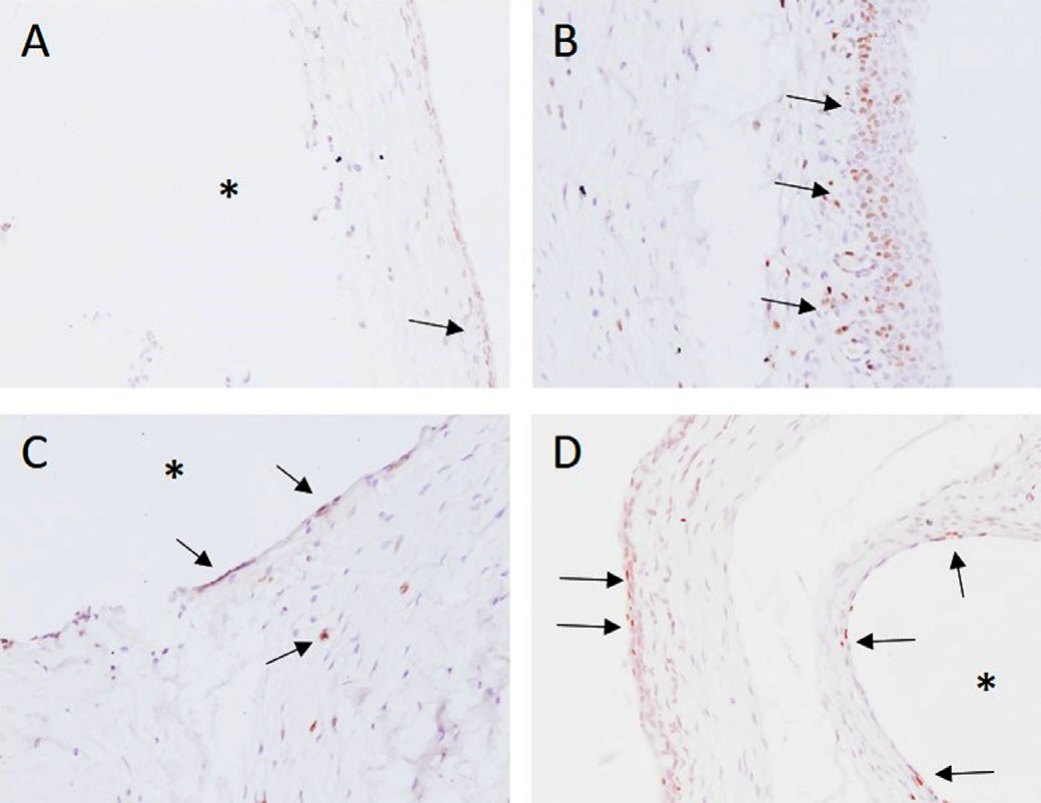
BrdU staining. (A) At POD 1, a limited amount of proliferation was noted in the conjunctiva (*arrow*), and no proliferation was seen around the implant site. (B) Increased proliferation was visible around the conjunctiva; additionally, the conjunctival layer was increased at POD 5. (C) A limited amount of proliferation was visible around the implant site at POD 5. (D) POD 40 revealed relatively low proliferation in the conjunctiva; however, around the implant site more proliferating cells were visible. *Position of SIBS microshunt. Images were taken at 200× magnification.

To assess the fibrotic response after implantation of the microshunt, several stainings were performed. The H&E stain (Fig. 5) revealed a high infiltration of PMNs at POD 1 (Fig. 6A). The total amount of PMNs decreased at POD 5, and more macrophages were present as compared to PMNs at this time point (Fig. 6B). By POD 40, the infiltration of inflammatory cells was drastically decreased. Nevertheless, low amounts of PMNs and macrophages were still present at POD 40, whereas more macrophages were present compared to PMNs (Fig. 6C). Increased neovascularization and vessel swelling were noted on POD 5 (Fig. 5C). Interestingly, a thickening of the conjunctival wall was evident at PODs 1 and 5 (Fig. 5D), increasing to a cellular thickness of approximately three or four cell layers compared to healthy controls, which had a cellular thickness of one. A capsule surrounding the shunt was visible at POD 40. Some foreign body giant cells (FBGCs) were noted sporadically around the shunt at POD 40 (Fig. 6E). Nonetheless, an open space between the conjunctival wall and the collagen deposition was still visible at POD 40. No myofibroblasts were present at POD 1 (Figs. 7A, 7B). At POD 5, a limited amount of staining was evident at the conjunctiva (Figs. 8C, 8D). The α-SMA stain revealed a large amount of myofibroblasts present around the conjunctival wall at POD 40 (Fig. 7E). Although myofibroblasts were also visible throughout the filtering bleb and around the shunt (Fig. 7F), they were mainly concentrated at the bleb wall.

The BrdU staining showed some proliferating cells in the conjunctiva at POD 1 (Fig. 8A). At POD 5, a high amount of proliferation was seen at the conjunctiva (Fig. 8B). Additionally, a high amount of neovascularization, as was previously noted with the H&E, was seen (data not shown). Furthermore, a limited amount of proliferation was seen around the microshunt (Fig. 8C). At POD 40, a low amount of proliferation was noted in the conjunctiva; however, around the microshunt, an increased proliferation was seen compared to POD 5 (Fig. 8D).

## Discussion

Despite current advances in the development of surgical techniques, new devices, materials, and antifibrotic therapies,^11^^,^^17^^–^^25^ fibrosis is still a critical determinant for success after glaucoma surgery.^8^ To obtain more knowledge regarding the fibrotic response, normotensive New Zealand White rabbits were implanted with SIBS MicroShunts. Previous research, where rabbits were implanted with SIBS microshunts, showed that bleb survival was extended up to 1 year, with limited signs of myofibroblasts and fibrosis.^11^ Although we did not find results as favorable regarding bleb survival, we did see a relatively low response of myofibroblasts, comparable to the previous study of Acosta et al.^11^ However, we did find FBGCs, macrophages, and PMNs surrounding the microshunt. With regard to bleb survival, similar results were found when compared to other studies, with bleb failure occurring around 2 weeks without the additional use of MMC or other antifibrotic agents.^26^^–^^28^ IOP results showed a trend similar to that for bleb survival, although no significant differences were found when compared to the healthy control eyes. IOP decreased at POD 1 and gradually began to increase again until POD 11. No large differences were observed between the control and operated eye after POD 11. Interestingly, the average IOP remained lower than the average IOP at baseline, suggesting that a residual amount of AqH might still flow into the bleb. Unfortunately, IOP is known to be an unclear outcome parameter with regard to glaucoma filtration surgery in animals. IOP deviations are often small within this type of animal model, as normotensive animals without glaucoma are used.^29^^,^^30^

Histology showed an increased amount of red blood cells around the surgical site, and PMNs were visible in high numbers in the bleb at POD 1. In general, PMNs are attracted due to damage-associated molecular patterns, leakage of plasma proteins, and chemokines released from thrombocytes or red blood cells.^16^^,^^31^ At POD 5, increased neovascularization, macrophage presence, and proliferation were noted. Although fibroblasts were clearly present, no high amounts of myofibroblasts were observed at POD 5. It was also observed that the bleb was further contracted at POD 40 as compared to the previous time points. At POD 40, a small capsule was visible around the SIBS shunt, with low amounts of myofibroblasts, fibroblasts, macrophages, and foreign body giant cells surrounding the implant. Additionally, in the bleb area, myofibroblasts were seen. Although in our histology specimens an open space was still visible inside the bleb, this was most likely an artifact caused by the manipulation of tissue during enucleation and processing of the eye.

As mentioned, inflammatory cells were present in high amounts at POD 1 (PMNs) and POD 5 (macrophages). Interestingly, in fetal wounds, wounds tend to heal without the formation of fibrotic tissue, which is thought to be attributable to the limited inflammatory response and reduced neutrophil and macrophage infiltration.^32^ Therefore, ameliorating or reducing PMN infiltration might offer a mechanism to reduce the fibrotic response, especially for glaucoma patients who have a predisposition for an increased inflammatory response due to the influx of AqH, which contains growth factors such as transforming growth factor β (TGF-β) and vascular endothelial growth factor.^33^^–^^35^ Recently, it was shown that inhibition of monocyte chemoattractant protein-1 (MCP-1) resulted in increased bleb survival and a downregulation of profibrotic genes. MCP-1 is known to be one of the primary proteins secreted by monocytes and macrophages to attract additional macrophages.^36^ Therefore, inhibition of proinflammatory cytokines or chemoattractants could offer a potential target for the downregulation of the wound-healing response in the bleb. These experiments suggest that a chronic state of inflammation is reached at POD 40, when some PMNs and macrophages are still present in the healed bleb area, which can predispose the tissue to an increased fibrotic response in the event a second surgery is performed (due to failure of the first surgery). Future research could try to elucidate which type of macrophages are present within the bleb at this time. The presence of M2 macrophages is necessary to resolve inflammation and begin the process of tissue remodeling.^37^ However, a persistent presence of M2 macrophages can predispose tissue to the formation of fibrosis.^38^ By altering or skewing the polarization state of macrophages to a more favorable state, such as reducing the amount of M2 macrophages, the fibrotic response could be reduced.

An increased amount of proliferation was noted at POD 5 in the conjunctival wall and inside the bleb, most likely due to neovascularization and fibroblast proliferation. Additionally, thickening of the conjunctival wall was noted. Epithelial cells are in a constant state of renewal, where new cells are being formed and older cells undergo apoptosis. However, potentially due to the increase of growth factors, such as TGF-β, it is likely that an increased proliferation is seen in these epithelial cells, increasing the thickness of the conjunctival wall. The increased presence of epithelial cells could also induce a different process referred to as epithelial mesenchymal transition (EMT). During EMT, epithelial cells, under the influence of TGF-β, transdifferentiate into mesenchymal stem cells and afterwards into fibroblasts.^39^ Additionally, a study by Potenta et al.^40^ showed that endothelial cells in the microvasculature can undergo a similar process known as endothelial mesenchymal transition (EndMT). Future research is required to confirm if conjunctival epithelial cells can also undergo EMT. If so, reducing EMT and EndMT might offer a potential therapeutic approach to alter the fibrotic response after glaucoma surgery. Further, if reducing EMT and EndMT is effective, perhaps a new line of antifibrotic agents could be developed such as bone morphogenic protein 7, which has been shown to reverse TGF-β–induced EMT.^41^ Currently, MMC is used during bleb-forming glaucoma surgery to reduce the fibrotic response.^9^ It is thought that MMC reduces the resident fibroblast response and reduces the number of fibroblasts that can transdifferentiate into myofibroblasts. As we observed in our study, myofibroblasts were predominantly present at POD 40 during the tissue remodeling phase. Myofibroblasts create the contractile forces required to contract a wound.^42^ Thus, it can be hypothesized that MMC, while still hampering the fibrotic response by inhibiting the proliferation and transdifferentiation of resident fibroblasts could also have an effect (either direct or indirect) on infiltrating cells such as PMNs and macrophages, as these are present early in the wound-healing response.

Novel methods to inhibit the formation of fibrosis would have to be safer than and as effective as MMC. Several approaches could be of interest for future research. First, antifibrotic therapies could be improved. Current novel antifibrotic therapies, although often safer in vivo, do not outperform MMC.^30^ Novel targets (as described earlier in this section) could be studied to find alternatives. Second, augmenting material properties could be beneficial. Implants innately induce a foreign body response, inducing encapsulation of the device/implant within the tissue. Our study shows that, although limited, a SIBS microshunt also triggers a foreign body response. Several ways exist to change the material properties, such as chemical modifications,^43^ coatings,^44^ plasma treatment,^45^ and alterations to surface topographical features.^46^^–^^48^ Surface topographies have received a lot of attention over the years in the medical field due to their ability to skew the reaction that cells have toward a material. For example, recent in vitro findings show that surface topographies can skew macrophage responses into M1 or M2 reactions.^49^ Moreover, it has been shown that surface topographies on breast implants are able to skew the encapsulation process in vivo.^50^ One study with large glaucoma implants such as the Ahmed glaucoma valves showed that a rough surface can increase the encapsulation of an implant, potentially caused by an increased adhesion of fibroblasts onto a rough surface or due to micromovements of Tenon's capsule and conjunctiva on the surface of the implant.^46^ It remains unclear which type of surface topographies can offer a more reliable outcome for glaucoma implants. Finally, the addition of a drug delivery system for sustained medication could reduce the fibrotic response, as low dosages of a drug can be released over an extended period of time. However, the type of drug, exact dosage, time of release, and material for the drug delivery system are currently unclear, and more research is needed.

## Conclusions

We observed that, after implantation of a SIBS microshunt in rabbits, a wide variety of cells are present during wound healing, such as monocytes/macrophages, PMNs, FBGCs, and epithelial cells. These cells offer new avenues for future research to develop novel methods to reduce the fibrotic response in vivo—for example, by developing novel antifibrotic drugs and novel methods for sustained drug delivery or by augmenting material properties.
